# Self‐Reported Attention to Positive Versus Negative Nutrients During Breakfast Cereal Selection Is Associated With Healthier Food Choice

**DOI:** 10.1111/nbu.70025

**Published:** 2025-08-06

**Authors:** Christopher R. Gustafson, Henriette Gitungwa, Devin J. Rose

**Affiliations:** ^1^ Department of Agricultural Economics University of Nebraska‐Lincoln Lincoln Nebraska USA; ^2^ Department of Food Science and Technology University of Nebraska‐Lincoln Lincoln Nebraska USA; ^3^ Department of Agronomy & Horticulture University of Nebraska‐Lincoln Lincoln Nebraska USA; ^4^ Nebraska Food for Health Center University of Nebraska‐Lincoln Lincoln Nebraska USA

**Keywords:** food choice, nutrition information, nutrition label use, nutrition policy

## Abstract

Nutritional guidance sometimes prioritises avoiding certain ‘negative’ nutrients over seeking out ‘positive’ nutrients (i.e., nutrients recommended to “limit” or “increase” in dietary guidelines). This study evaluated the association between attention to positive or negative nutrients and the nutritional quality of ready‐to‐eat breakfast cereal choices. In an online survey, 962 adult US residents chose from 33 cereals displayed with nutritional information (i.e., energy, fat, sugar, sodium (negative), fibre, potassium, iron [positive]). After choosing, participants reported use of nutritional information. A “Guiding Stars” (GS) rating, which categorises foods based on their nutrient contents (not displayed to panellists during food choice), was used to categorise the healthfulness of cereals. Ordinal logistic regression was used to determine the relationship between attention to positive or negative nutrients and the GS rating of cereal choices. Each positive nutrient considered increased the odds of selecting a cereal with a higher GS rating 2.83 (95% confidence interval: 2.13, 3.80) times compared with someone considering no nutrients, which was significantly higher (*p* = 0.0015) than each additional negative nutrient considered (1.55 [1.33, 1.82]). For people considering only negative nutrients, the odds of selecting a cereal with a higher GS rating was 2.55 (1.89, 3.47) times that of someone considering no nutrients, but was 6.76 (3.72, 12.58) or 8.86 (6.09, 13.03) for people that considered only positive or both positive and negative nutrients. Thus, nutritional recommendations that highlight the importance of considering positive nutrients may be more effective at increasing the nutritional quality of food choices than messages focused on avoidance of negative nutrients.

## 1 Introduction

In the US and many other countries, consumers are provided with nutritional information on food packages. This information can be on the side or back of the package in an information panel or on the front of the package (i.e., Front‐of‐Pack (FOP) labels) (Donini et al. 2022; Dumoitier et al. 2019). Estimates of the exact proportion of consumers that view or use nutritional information during food choice vary widely (16%–90%) by product type and method of data collection (Graham and Jeffery 2012; Grebitus and Davis 2017; Grunert, Fernández‐Celemín, et al. 2010; Grunert, Wills, and Fernández‐Celemín 2010; Todd and Variyam 2008; Visschers et al. 2010). However, attention to nutritional information during food choice is essential to making informed, healthy food decisions that are in line with dietary recommendations (Barreiro‐Hurlé et al. 2010; Grebitus and Davis 2017). Therefore, many studies have focused on strategies to increase consumers' attention to nutritional information (Arslain et al. 2021; Barreiro‐Hurlé et al. 2010; Bix et al. 2015; Cowburn and Stockley 2005; Donini et al. 2022; Graham and Jeffery 2012; Grebitus and Davis 2017; Grunert, Fernández‐Celemín, et al. 2010; Grunert, Wills, and Fernández‐Celemín 2010; Ladeira et al. 2023; Storcksdieck Genannt Bonsmann et al. 2020).

Demographic variables affect whether a person is prone to look at nutritional information. In an analysis of Swiss participants, those that looked at nutrition information tended to be women with excess weight that likely followed a special diet in the past year (Visschers et al. 2013). Women also tended to use nutrition information during food choice in Malaysia. These women were primarily single, employed, physically active 18–30‐year‐old non‐smokers (Cheah et al. 2015). In a Croatian population, use of nutrition information was not different between men and women, but was highest among college‐educated 30–44‐year‐olds with high degrees of nutritional knowledge (Krešić and Mrduljaš 2016). In people with chronic diseases in the US, nutritional knowledge was a more important factor in predicting nutrition label use than age, gender, race, chronic disease condition, and caregiver status (Rose et al. 2018). Among adults aged 20 and older who completed the National Health and Nutrition Examination Survey (NHANES), those who were trying to lose weight were most likely to use nutritional information (Bleich and Wolfson 2015).

Among consumers that view or use nutritional information on food packages, there is variation in the specific nutrients that are used. Among these nutrients, fat, sugar, dietary fibre, and sodium appear to be the most commonly considered (Goldberg et al. 1999; Graham and Jeffery 2011; Marietta et al. 1999; Misra 2007; Todd and Variyam 2008).

Given the rise in diet‐related diseases, much of the nutritional guidance popular media provided over the past decades has focused on avoiding certain foods or nutrients that have been associated with disease, as opposed to seeking foods high in beneficial nutrients (Wahl et al. 2017). However, restrictive diets that emphasise avoiding certain nutrients or foods can often be counterproductive and lead to weight fluctuations or weight gain (Mann et al. 2007; Van Strien et al. 2014).

Official dietary guidance in the USA is outlined in the Dietary Guidelines for Americans (DGA) (US Department of Agriculture and US Department of Health and Human Services 2020). In the DGA, certain nutrients are recommended to be limited in the diet because they are associated with negative health outcomes and are generally over‐consumed by the public. These include saturated fat, added sugars, and sodium. The DGA also recommend staying within energy (kcal) limits because “meeting food group recommendations—even with nutrient‐dense choices—requires most of a person's daily calorie needs and sodium limits.” (US Department of Agriculture and US Department of Health and Human Services 2020). In contrast, other nutrients are recommended to be increased in the diet because they are associated with positive health outcomes and are generally under‐consumed by the public. The nutrients specifically recommended are dietary fibre, vitamin D, calcium, iron, and potassium.

Among the nutrients highlighted in the DGA, a previous study of US primary shoppers found that consumers focused more on the negative nutrients to avoid—energy, saturated fat, added sugars, and sodium—rather than the positive nutrients that are under‐consumed—dietary fibre, calcium, potassium, and vitamin D—when choosing what foods to buy or eat (Gustafson and Rose 2023). While this finding is interesting when considering how this might influence consumers' relationships with food, it is unknown whether attention to positive versus negative nutrients is associated with the healthfulness of food choices. Therefore, the objective of this study was to evaluate the association between consumer attention to positive or negative nutrients and the nutritional quality of food choices. For simplicity, nutrients that are positively associated with diet‐related metabolic diseases and the consumption of which people are recommended to limit in many dietary guidelines are referred to as “negative” nutrients, while those that are positively associated with healthful outcomes and the consumption of which people are recommended to increase in dietary guidelines are referred to as “positive” nutrients. We hypothesised that respondents who paid attention to more positive nutrients versus negative nutrients would make more healthful food choices, since avoidance diets, which can be equated to avoiding certain nutrients, generally result in poorer diet quality (Mann et al. 2007).

## 2 Methods

### 2.1 Survey

An online food choice scenario and survey was developed in Qualtrics (www.qualtrics.com), which is a widely used survey platform, to examine the relationship between the use of nutrient information and the nutritional value of food choice. The survey was distributed using the online platform, Prolific (www.prolific.com), to a convenience sample of adult (≥ 19 years old) residents of the USA. Participants received $2 for completing the study. The study was conducted in accordance with the Declaration of Helsinki and approved by the Institutional Review Board of the University of Nebraska‐Lincoln (IRB protocol #20201020721EX). All participants provided electronic informed consent before participating in the research.

The food choice survey consisted of three parts (see Supporting Information). The first part was a baseline food choice task in which participants selected a ready‐to‐eat breakfast cereal from a list of 33. The second part was an intervention scenario designed to test the effects of simple and educational prompt messages on participants' cereal choices. In the last part, respondents answered survey questions about food choice and demographic variables. This article reports data only from the baseline food choice task and the survey. Results from the intervention portion of the study were published previously (Gitungwa et al. 2024).

**FIGURE 1:**
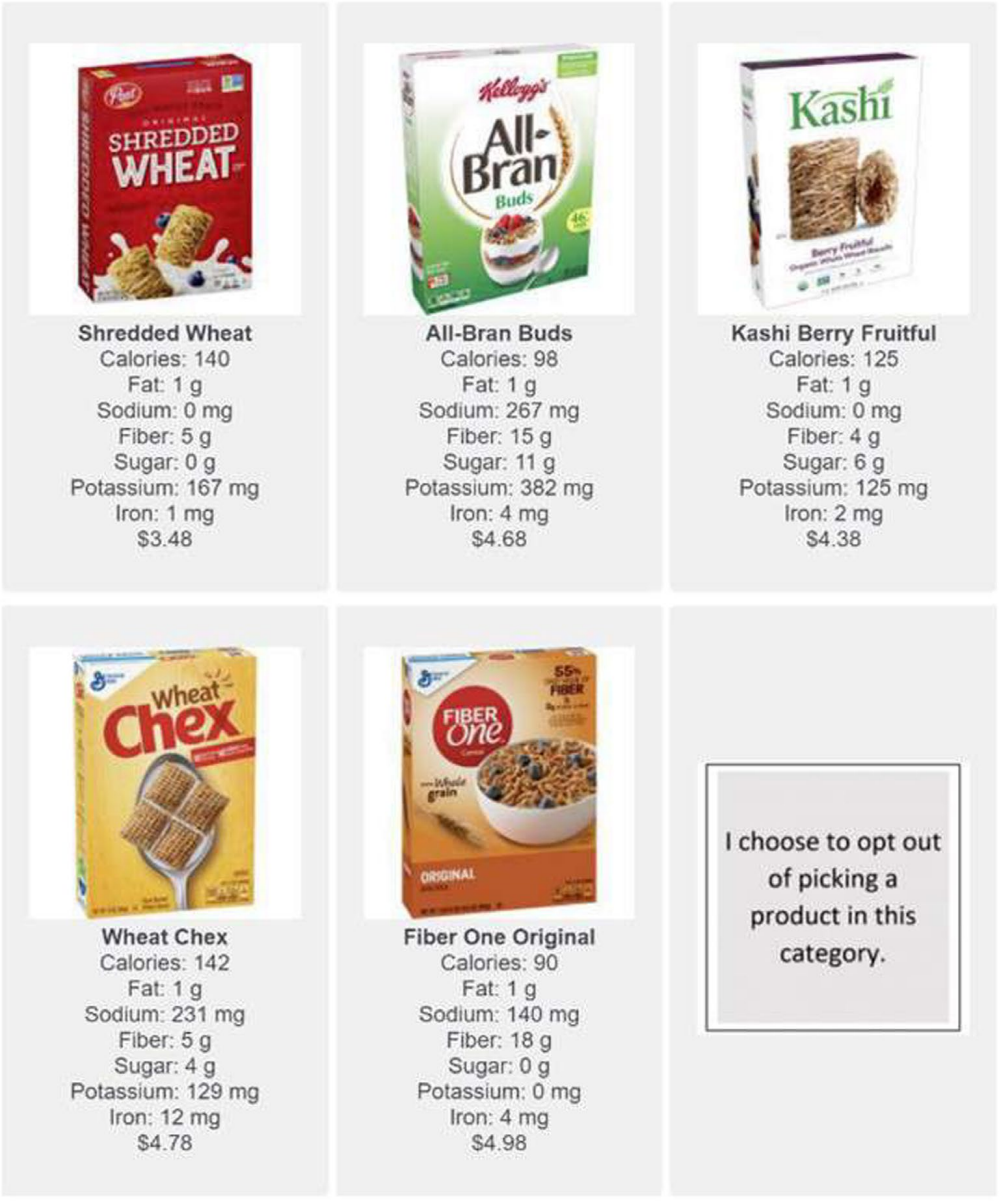
Screen shot of a portion of the cereal choice environment. Respondents selected a cereal from 33 options with an image of the cereal, nutrition information, and price; an option to not select any of the cereals was also included at the end.

The cereals that participants selected from were from national brands that are widely available at regional and national supermarket chains in the USA and represented a range of taste and nutrient profiles. When viewing cereals, information about the product was displayed below the image of the product, including the product's name; brand; per‐serving content of calories, fat, sodium, dietary fibre, sugar, potassium, and iron; and price (Figure 1). Importantly, the Guiding Stars (GS) rating for each cereal option, which was used in data analysis to assess healthfulness of cereals and is described further in the next section, was not provided to participants. Nutritional information was standardised to a 40 g serving size, which is the ‘Reference Amount Customarily Consumed Per Eating Occasion’ used on Nutrition Facts Panels in the USA (US Food and Drug Administration 2025), to make it easy for participants to compare among breakfast cereals and to avoid having to list both the nutrient amount and percent of recommended daily intake. The nutrients displayed were chosen because they are highlighted in the Dietary Guidelines for Americans as being nutrients to limit or nutrients that are of public health concern because their intake is below recommendations (US Department of Agriculture and US Department of Health and Human Services 2020) or because they were identified as nutrients that influence consumer's decisions about whether a food is “healthy” or “unhealthy” (Lusk 2019). Energy, fat, sodium, and sugar were considered “negative nutrients” and dietary fibre, potassium, and iron were considered “positive nutrients”. The cereal prices were based on retail prices at regional and national retailers at the time the survey was conducted. The time that a participant took to make a cereal choice was recorded. After the choice, participants answered survey questions about the use of nutritional information and demographics. In other research, questions about behaviours, such as use of nutrition information, that would influence the choice process if asked while the participant was making a decision have been asked after completion of the choice process (Morris et al. 2021) and found to correlate highly with objective measures collected using eye‐tracking (Machín et al. 2023). To make sure that participants were paying attention, a question in which participants were asked to mark “Added Sugar” from a list of six options was included. All participants marked the correct response to this question.

### 2.2 Categorising the Healthfulness of Cereals

The healthfulness of the cereals was categorised using the GS rating system (Fischer et al. 2011), which is a shelf‐based labeling system that categorises foods based on their overall healthfulness and is used widely in grocery stores in the eastern USA. In the GS rating system, foods are assigned zero (least healthy) to three (most healthy) stars based on the nutrient content. Considered in the GS algorithm are nutrients to avoid (*trans* fat, saturated fat, cholesterol, added sugar, and added sodium) and nutrients to encourage (dietary fibre, vitamins and minerals, and omega‐3 fats). Foods receive negative points if they reach certain values for nutrients to avoid and receive positive points if they reach certain values for nutrients to seek out per serving. Among the 33 ready‐to‐eat breakfast cereals available to respondents, 11 had zero GS, 11 had one GS, and 11 had two or three GS. Fewer cereals had three GS due to the limited number of cereals meeting the requirements for this category. Therefore, cereals with GS of two or three were combined into one group. The GS rating of cereals was not available to respondents at the time of cereal choice; only nutrient information (and price) was provided, as shown in Figure 1.

### 2.3 Data Analysis

All data were analysed using R (version 4.3.1) and RStudio (release b51c81cc) with various packages as noted (R Core Team 2022). Basic statistics were calculated using base R and the ‘rstatix’ package (Kassambara 2020). Of the 1002 respondents that completed the survey, 962 responses with complete data were used in the data analysis (excluded: 10 that indicated they would not choose any of the available cereals and 30 who preferred not to report income and/or education information). To estimate the association between attention to positive or negative nutrients and the nutritional quality of food choices, two ordinal logistic regression models were run using the ‘polr’ function in the ‘MASS’ package (Venables and Ripley 2002). In each model, the GS rating of the cereal chosen was the dependent variable. In the first model, the numbers of positive and negative nutrients each respondent reported using during cereal choice were calculated. These two numeric variables were the independent variables in the model. In the second model, respondents were categorised into groups based on their attention to nutrient information: consideration of no nutrients, consideration of only positive nutrients, consideration of both positive and negative nutrients (balanced), and consideration of only negative nutrients. Demographic variables—age, sex, race, education, and household income—were also included in both models. Linear hypothesis testing of effect sizes from the regression and ordinal logistic regression models was calculated using the ‘car’ package (Fox and Weisberg 2019). Predicted probabilities and standard errors from the logistic regression models were calculated using the ‘marginaleffects’ package (Arel‐Bundoc 2024). When predicted probabilities were calculated, the values for all other variables in the model except for the one being predicted were set at their reference values. Linear regression was used to examine the relationships between (1) the nutrients considered and nutrient content of cereal choices; (2) the time it took to make a cereal decision and the number of nutrients a participant reported considering; and (3) the number of nutrients considered and perception of the healthfulness of eating habits or participants' satisfaction with their current health status. Linear regression was performed using the ‘lm’ function in base R. Formatted statistical results tables were exported using the ‘stargazer’ package (Hlavac 2022). Plots were generated using the ‘ggplot2’ and ‘cowplot’ packages (Wickham 2016; Wilke 2020).

## 3 Results

### 3.1 Descriptive Statistics

Respondent demographics (age, sex, race, education, and household income) are presented in Table 1. The majority of respondents were 25–44 years old (58%), female (52%), white (81%), and college‐educated (83%), with household incomes > $40 000/year (66%).

**TABLE 1:** Demographics of study participants.

Variable	*N*	Percent
Age		
19–24 years	148	15%
25–34 years	315	33%
35–44 years	248	26%
45–54 years	110	11%
55–64 years	94	10%
65 years and older	47	5%
Sex		
Not female	452	47%
Female	510	53%
Race		
White	783	81%
Asian	61	6%
Black or African American	64	7%
Latino	44	5%
Others or prefer not to answer	10	1%
Education		
High School	155	16%
College	807	84%
Income		
< $20 000	107	11%
$20 000–$39 999	198	21%
$40 000–$59 999	170	18%
$60 000–$79 999	171	18%
$80 000–$99 999	101	10%
> $100 000	215	22%
Total	962	100%

### 3.2 Nutritional Content of Cereal Choices

**FIGURE 2:**
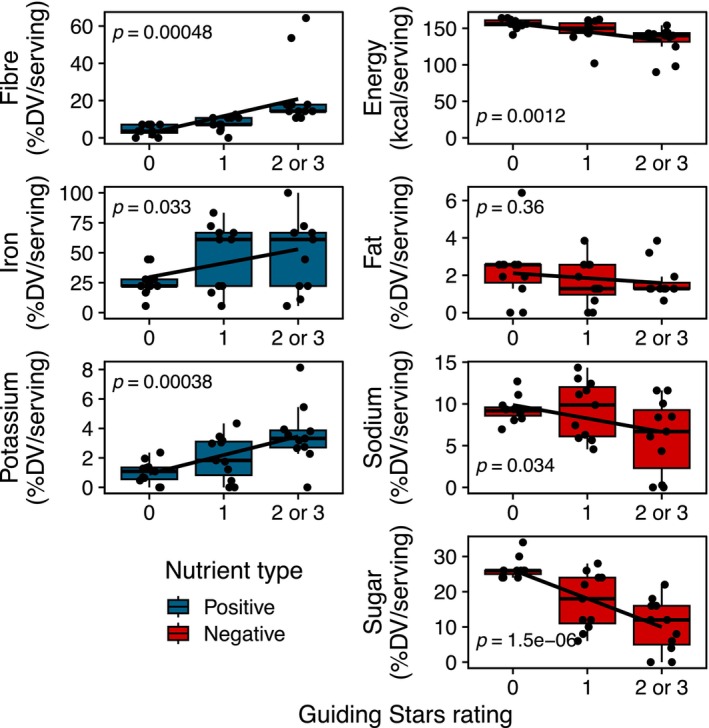
Nutrient content of cereal choices by Guiding Stars rating. *N* = 11 cereals/Guiding Stars rating; DV, daily value; 1 serving = 40 g; *p*‐values shown correspond to the slope of the linear relationship between Guiding Stars rating and nutrient content.

The nutritional information and GS rating of all 33 cereal choices available to participants is reported in Table S1. To determine the usefulness of the GS rating at expressing the overall healthfulness of cereals, the associations between nutrient contents of each cereal available to participants in this study and GS rating were determined. As shown in Figure 2, the GS rating captured the overall healthfulness of cereals, with decreasing concentrations of the negative nutrients, sodium and added sugar, and increasing concentrations of the positive nutrients, dietary fibre, iron, and potassium, as GS rating increased. Only fat, among the negative nutrients, did not show a significant negative trend as GS rating increased. Cereals with a GS rating of zero were chosen by 41% of respondents, while cereals with GS ratings of one or two or three (two or three were combined into one category) were about evenly split at 30% and 29%, respectively (Table 2).

**TABLE 2:** Summary of Guiding Stars (GS) rating of cereal choices and consideration of nutritional information.

Variable	*N*	Percent
GS rating of cereal choice		
0	394	41%
1	288	30%
2 or 3	280	29%
Use of nutritional information		
No nutrients considered	542	56%
Considered fibre	181	19%
Considered iron	39	4%
Considered potassium	33	3%
Considered energy	211	22%
Considered fat	59	6%
Considered sodium	65	7%
Considered sugar	265	28%
Category of nutrient types considered		
None	542	56%
Only positive	45	5%
Positive and negative	160	17%
Only negative	215	22%

### 3.3 Use of Nutritional Information During Cereal Choice

More than half of respondents (*n* = 542, 56%) did not consider any nutritional information, while *n* = 420 (44%) considered some nutritional information during food choice (Table 2). The most commonly considered nutrients were sugar (*n* = 265) and fibre (*n* = 181), with few respondents considering iron (*n* = 39) and potassium (*n* = 33). Respondents considered more negative nutrients than positive, with a total of 215 participants considering only negative nutrients and only 45 considering only positive nutrients.

**FIGURE 3:**
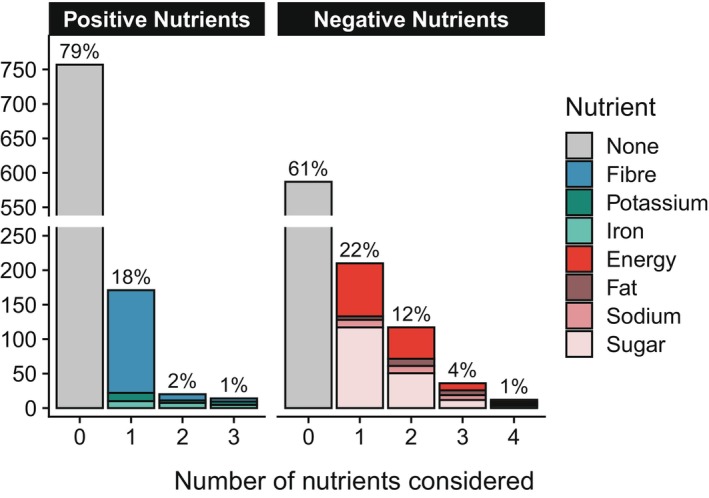
Use of nutrients by nutrient type during cereal choice. Use of nutritional information by nutrient type and the number of nutrients selected.

Nearly 80% of respondents did not consider any positive nutrients, while just over 60% of respondents did not consider any negative nutrients (Figure 3). If only one positive nutrient was selected, it tended to be fibre. The other positive nutrients, iron and potassium, were considered by a minority of panellists regardless of the number of positive nutrients they considered. Of those that considered negative nutrients, energy and sugar were most commonly considered and represented the majority of first and second negative nutrients considered.

### 3.4 Relationship Between the Number and Type of Nutrients Considered in Cereal Choice and Healthfulness of Cereal Choice

For each positive nutrient considered during cereal choice, the odds of selecting a cereal with a higher GS rating increased by a factor of 2.83 relative to no nutrients considered (Table 3). The odds of selecting a cereal with a higher GS rating also increased for each negative nutrient considered, but only by a factor of 1.55 compared with no nutrients considered. The odds ratio for positive nutrients was significantly greater than that for negative nutrients (*p* = 0.0015).

**TABLE 3:** Ordinal logistic regression on the influence of the number of positive or negative nutrients used during decision‐making and demographic variables on Guiding Stars rating of cereal choices.

Variable	OR	(95% CI)	*p*
Number of nutrients considered (reference: none)			
Positive^a^	2.83	(2.13, 3.8)	< 0.001
Negative	1.55	(1.33, 1.82)	< 0.001
Age (reference: 19–24 years)			
25–34 years	1.59	(1.07, 2.39)	0.023
35–44 years	2.47	(1.63, 3.78)	< 0.001
45–54 years	3.22	(1.97, 5.31)	< 0.001
55–64 years	3.81	(2.25, 6.51)	< 0.001
65 years and older	7.81	(3.91, 16.06)	< 0.001
Sex (reference: not female)			
Female	1.41	(1.09, 1.82)	0.009
Race (reference: white)			
Asian	1.14	(0.67, 1.94)	0.63
Black or African American	0.80	(0.48, 1.31)	0.37
Latino	0.95	(0.52, 1.72)	0.87
Others or prefer not to answer	1.03	(0.28, 3.56)	0.96
Education (reference: high school)			
College	1.21	(0.85, 1.73)	0.29
Income (reference: < $20 000)			
$20 000–$39 999	1.47	(0.91, 2.38)	0.12
$40 000–$59 999	1.57	(0.96, 2.58)	0.073
$60 000–$79 999	1.19	(0.73, 1.96)	0.49
$80 000–$99 999	1.46	(0.84, 2.53)	0.18
> $100 000	1.72	(1.07, 2.78)	0.025

Abbreviations: CI, confidence interval; OR, odds ratio.

^a^ Linear hypothesis test of positive–negative = 0, *p* = 0.0015.

Certain demographic characteristics also influenced the healthfulness of cereal choices. In particular, the older a participant was, the healthier their cereal choices tended to be (Table 3). Females also chose healthier cereals compared with non‐females by a factor of 1.41. Race and education did not affect the healthfulness of cereal choices, but participants in the very highest income bracket (> $100 000/year) made significantly healthier cereal choices than people in the lowest income bracket (< $20 000/year).

**FIGURE 4:**
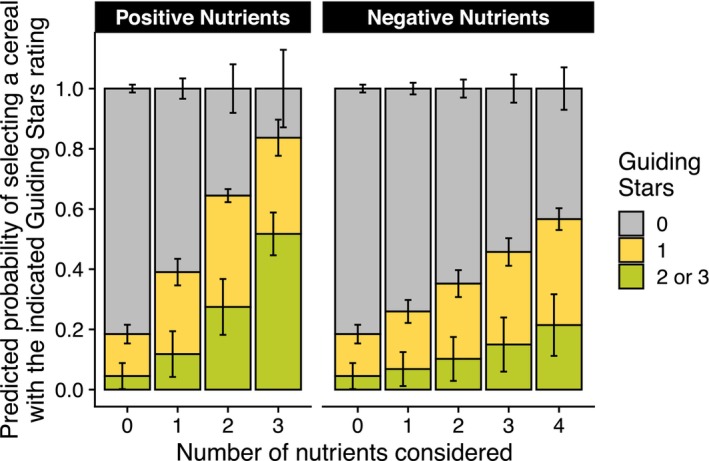
Predicted healthfulness (Guiding Stars rating) of cereal choices depending on the number of negative or positive nutrients considered during the choice process. Predicted probabilities calculated from ordinal logistic regression where the number of positive or negative nutrients used during decision‐making, after adjusting for age, sex, race, education, and income, was regressed on Guiding Stars rating of cereal choices.

The impact of considering positive nutrients over negative nutrients is best visualised in a predicted probabilities plot (Figure 4). Here, the demographic variables were set to their reference values and the predicted probabilities of selecting cereals at each GS rating were calculated. The predicted probability of selecting a cereal with a GS rating of one or more was only 0.18 for respondents that considered no nutritional information. This probability increased to 0.26, 0.35, 0.48, and 0.57 when considering one to four negative nutrients, respectively, but increased much more—to 0.39, 0.64, and 0.84—for one, two, or three positive nutrients.

### 3.5 Relationship Between the Category of Nutrients Considered in Cereal Choice and Healthfulness of Cereal Choice

When respondents were categorised by the type of nutrients they predominantly attended to during food choice, attention to only positive nutrients increased the odds ratio of selecting a cereal with a higher GS rating by a factor of 6.76 compared with no nutrients considered (Table 4). In contrast, for respondents that focused only on negative nutrients, the odds ratio only increased by a factor of 2.55 compared with no nutrients considered. The odds ratio for considering only positive nutrients was significantly greater than that of negative nutrients (*p* = 0.0025). The odds ratio of selecting a cereal with a higher GS rating for respondents that considered both positive and negative nutrients was 8.86 compared with no nutrients considered, which was not significantly different from the only positive nutrients group (*p* = 0.42).

**TABLE 4:** Ordinal logistic regression of the attention to more positive, more negative, or both positive and negative nutrients during decision‐making on Guiding Stars rating of cereal choices (reference: no nutrients considered).

Variable	OR	(95% CI)	*p*
Category of nutrient types considered (reference: no nutrients considered)			
Only positive nutrients considered^a^	6.76	(3.72, 12.58)	< 0.001
Positive and negative nutrients (both)	8.86	(6.09, 13.03)	< 0.001
Only negative nutrients considered	2.55	(1.89, 3.47)	< 0.001
Age (reference: 19–24 years)			
25–34 years	1.54	(1.03, 2.32)	0.037
35–44 years	2.53	(1.66, 3.87)	< 0.001
45–54 years	3.04	(1.86, 5.02)	< 0.001
55–64 years	3.90	(2.29, 6.69)	< 0.001
65 years and older	7.31	(3.62, 15.21)	< 0.001
Sex (reference: not female)			
Female	1.45	(1.12, 1.88)	0.0045
Race (reference: white)			
Asian	1.21	(0.71, 2.05)	0.48
Black or African American	0.83	(0.5, 1.36)	0.46
Latino	0.88	(0.48, 1.6)	0.68
Others or prefer not to answer	0.99	(0.26, 3.49)	0.99
Education (reference: high school)			
College	1.19	(0.84, 1.71)	0.33
Income (reference: < $20 000)			
$20 000–$39 999	1.43	(0.89, 2.33)	0.14
$40 000–$59 999	1.57	(0.96, 2.57)	0.075
$60 000–$79 999	1.15	(0.7, 1.9)	0.57
$80 000–$99 999	1.35	(0.78, 2.34)	0.29
> $100 000	1.65	(1.03, 2.67)	0.038

Abbreviations: CI, confidence interval; OR, odds ratio.

^a^ Linear hypothesis test of Positive–Both = 0, *p* = 0.42; Positive–Negative = 0, *p* = 0.0025; Both–Negative = 0, *p* < 0.001.

The same demographic characteristics had significant influences on the healthfulness of cereal choices when respondents were categorised by the type of nutrients they predominantly attended to during food choice as when the number of nutrients used was calculated. Specifically, age, sex, and income had significant effects on the GS rating of cereal choices, with older, female, and more affluent participants selecting healthier cereals.

**FIGURE 5:**
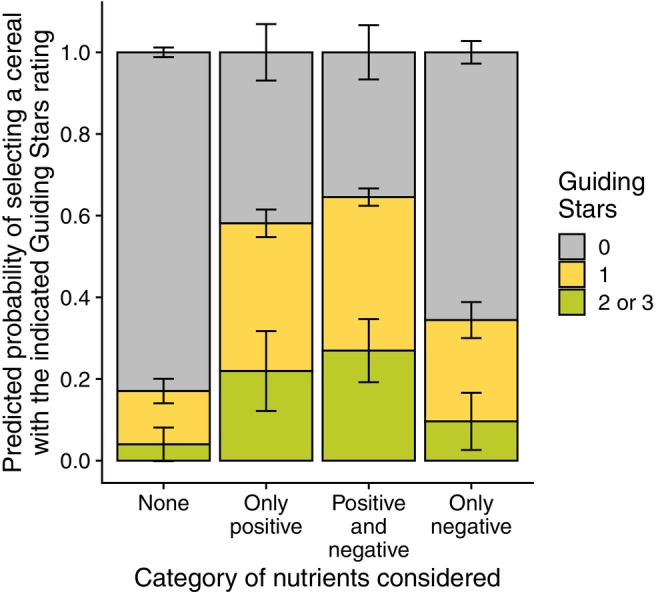
Predicted healthfulness (Guiding Stars rating) of cereal choices depending on the type of nutrients considered during the choice process. Predicted probabilities calculated from ordinal logistic regression where the category of nutrients considered during decision‐making, after adjusting for age, sex, race, education, and income, were regressed on Guiding Stars rating of cereal choices.

These results were also visualised in a predicted probabilities plot, where demographic variables were again set to their reference values (Figure 5). The predicted probability of selecting a cereal with a GS rating of one or more was 0.17 for respondents that considered no nutrients during cereal choice. This probability increased to 0.34 for respondents that considered only negative nutrients but increased to 0.65 and 0.58 for those that considered both positive and negative nutrients or only positive nutrients, respectively.

### 3.6 Time Burden of Considering Nutrients During Cereal Choice

Because considering nutritional information during cereal choice would presumably require more cognitive investment for participants, we analysed the time it took to make a cereal choice based on the number and type of nutrients considered (Table 5). On average, respondents that considered no nutrients took slightly more than 36 s to make a cereal choice. For each additional negative nutrient considered, an average of 6.24 additional seconds was required to make a cereal choice, while an additional 7.08 s was required for each positive nutrient considered. The added time required to make a cereal choice for each additional nutrient was not significantly different between negative and positive nutrients (*p* = 0.81).

**TABLE 5:** Linear regression of the number of healthy or unhealthy nutrients considered on the time (seconds) it took to make a cereal choice.

Variable	Estimate^a^, ^b^	(SE)	*p*
Number of positive nutrients considered	7.08	(2.54)	0.0053
Number of negative nutrients considered	6.24	(1.56)	< 0.001
Intercept	36.3	(5.39)	< 0.001

^a^ Linear regression estimates adjusted for age, sex, race, education, and income.

^b^ Linear hypothesis test of Positive–Negative = 0, *p* = 0.81.

### 3.7 Relationship Between Specific Nutrients Considered and the Content of Those Nutrients in Cereal Choice

Although we were primarily interested in how consideration of negative versus positive nutrients affected the overall healthfulness of cereal choices, consideration of nutrients was self‐reported by respondents. Therefore, we analysed the relationship between self‐reported consideration of specific nutrients and the content of those nutrients in cereal choices (Table 6). For five out of the seven nutrients considered in this study, significant relationships were found between reported consideration of those nutrients during decision making and the content of that nutrient in the cereal chosen by the participant. The two nutrients that did not show significant linear trends had relatively low variance in that nutrient among cereals and were borderline significant (fat, *p* = 0.05) or had relatively few respondents that considered that nutrient (iron, *n* = 39). Regardless of significance, the direction of the relationship—negative for negative nutrients and positive for positive nutrients—was as expected.

**TABLE 6:** Linear regression of the consideration of specific nutrients during decision‐making on nutrient content (per 40 g serving) of cereal choices.

Variable	Estimate^a^	(SE)	*p*
Considered energy^b^	−6.37	(0.98)	< 0.001
Considered fat	−0.44	(0.23)	0.052
Considered sodium	−2.03	(0.44)	< 0.001
Considered sugar	−4.48	(0.58)	< 0.001
Considered fibre	7.81	(0.70)	< 0.001
Considered iron	6.31	(4.22)	0.14
Considered potassium	0.58	(0.27)	0.029

^a^ Linear regression estimates adjusted for age, sex, race, education, and income.

^b^ Units for energy are kcal; units for other nutrients are % daily value.

### 3.8 Relationship Between Participants' Perceived Health Status and Eating Habits and the Number and Type of Nutrients Considered in Cereal Choice

We asked questions about the panellists' perceived health status and eating habits in an attempt to explain philosophies associated with nutrient consideration during cereal choice. When asked about their perception of the healthfulness of their eating habits, those that indicated their eating habits were ‘healthy’ considered 0.10 (standard error: ±0.05, *p* = 0.01) more positive nutrients and 0.31 (±0.07, *p* < 0.001) more negative nutrients during decision‐making than those who thought their eating habits were ‘neither healthy nor unhealthy’ or did not know about the healthfulness of their eating habits. In contrast, those that indicated their eating habits were ‘unhealthy’ did not consider more positive (−0.06 ± 0.06, *p* = 0.26) or negative (−0.03 ± 0.09, *p* = 0.72) nutrients during decision‐making than the ‘neither healthy nor unhealthy’ or ‘I do not know’ panellists. The number of positive or negative nutrients considered during cereal choice was not related to the panellists' satisfaction with their current health status (*p* > 0.05), likely because the question did not distinguish between people who were actually healthy and thus satisfied with their current health status, and those that reported being satisfied with their health status because they place low value on health.

## 4 Discussion

Our study showed that attention to nutrition information is associated with self‐perceived healthy eating habits, aligning with prior research on the role of nutrition information in food choices (Barreiro‐Hurlé et al. 2010; Grebitus and Davis 2017). Therefore, many studies have focused on new ways to present nutritional information that will lead to greater attention to nutritional information; however, few studies have examined how the types of nutrients consumers pay attention to during food choice affect the healthfulness of food choice. Our hypothesis that respondents who paid more attention to positive nutrients versus negative nutrients would make more healthful food choices was confirmed. Indeed, those that considered only positive nutrients in their cereal choice were more than twice as likely to select a cereal with a higher GS rating than those that focused only on negative nutrients. Thus, developing strategies that help consumers focus on the positive nutrients in foods may have a greater impact on increasing the healthfulness of consumers' food choices than directing them to nutrients to avoid.

There is some emerging evidence that prompts to consider nutrients, such as dietary fibre, delivered at the beginning of a food choice event both increase use of fibre information during food choice, increase the amount of dietary fibre per serving of foods chosen, and increase the overall nutritional quality of foods selected (Arslain et al. 2020, 2021; Gustafson 2023). Several authors have noted that nutritional messages should focus on whole foods, rather than individual nutrients, since people eat foods not nutrients (Donini et al. 2022). Thus, a message suggesting consumption of more pulses because they are excellent sources of fibre, for example, may be a better message than simply instructing respondents to eat more fibre (Gustafson et al. 2024). The effect of such messages is worthy of future investigation.

Importantly, there was a group of respondents that focused on both positive and negative nutrients during cereal choice. These respondents were similar to those that focused only on positive nutrients in terms of the healthfulness of the cereal choices they made. Thus, there is a place for negative nutrients; they should just not be the focus.

Forty‐four percent of the study population reported using nutrition information during cereal choice. This is similar to the self‐reported percentage of nutrition information usage in the US (39%) (Grebitus and Davis 2017) and somewhat higher than that reported in the UK (27%) (Grunert, Wills, and Fernández‐Celemín 2010). It is lower than eye‐tracking approaches, which report > 60% usage of the nutrition information, although they only indicate if a subject looked at the nutritional information, not if it was used in the decision‐making process (Graham and Jeffery 2012; Visschers et al. 2010). Eye‐tracking studies also tend to have participants consider small numbers of products—typically only a few at a time (Bialkova et al. 2014). Increasing the number of product options and/or attribute numbers reduces attention to individual products and information (Meißner et al. 2020).

Consumers that reported using nutrition information during cereal choice tended to focus on negative nutrients over positive. This is consistent with a previous report of a large sample of primary USA shoppers (Gustafson and Rose 2023). This is likely because nutritional guidance and popular weight loss diets often focus on avoiding certain foods and nutrients (Wahl et al. 2017). Thus, consumers are likely influenced by these messages and focus on what to avoid rather than what to seek out. Constantly looking at food as a source of nutrients to avoid may have negative health consequences. Indeed, restrictive dieting to reduce body weight can have unintended effects of promoting guilty feelings, increasing weight gain, and distorting body image (Broers et al. 2021; Mann et al. 2007; Van Strien et al. 2014). In contrast, people who defined healthy foods as being rich in vegetables and made at home (positive thinking) ate higher (self‐reported) quality diets than people that thought of healthy foods as inconvenient, expensive, and undesirable (negative thinking) (Yarar and Orth 2018).

In addition to the type of nutrients considered, certain demographic variables affected the healthfulness of cereals chosen. In particular, age had the most pronounced effect on healthfulness, with older participants selecting healthier cereals. Female and more affluent respondents also tended to select healthier cereals than non‐females and less affluent participants. Others have reported similar findings (Cheah et al. 2015; Visschers et al. 2013).

Our study examined associations between nutrient use and the nutritional quality of food choice among consumers that indicated they considered nutritional information of their own volition. It remains to be seen whether interventions that direct these consumers to positive nutrients will improve the nutritional quality of food choices compared with interventions that direct attention to negative nutrients or nutrients in general. As noted previously, there is some evidence that a prompt to consider dietary fibre—a positive nutrient—delivered at the beginning of food choice can increase the amount of fibre per serving in the foods selected by increasing the use of fibre information, which significantly mediates the choice of higher‐fibre products (Arslain et al. 2020, 2021; Gustafson 2023). Whether this can be used for other nutrients and if messages about positive nutrients are more effective than messages about negative nutrients remains to be seen. Future studies should test interventions that prompt these consumers to look at the positive versus negative nutritional aspects of foods to examine the impact on the nutritional quality of these consumers' food choices. These are important areas of future research.

A limitation of our study is that product choices were hypothetical. Thus, participants may not have invested as much effort in considering cereal options or reading nutrition information as they would have if the choices were real. We attempted to address this potential bias by prompting participants to approach the choice as if they were going to receive the product and spend money, which has been found to reduce hypothetical bias (Lusk 2003). We also included an attention check question to make sure that participants were paying attention.

Another limitation is that consideration of nutritional information was self‐reported. Although our results are similar to previous reports using self‐reported data (Grebitus and Davis 2017), self‐reported data are subject to social‐desirability bias, where respondents may indicate that they used nutritional information because they perceive that it is the more socially desirable response (Visschers et al. 2010). One way that we addressed this limitation was to analyse the relationship between respondents' self‐reported nutrient consideration and the nutrient contents of the cereals they chose. This analysis supported respondents' answers, as those that indicated considering negative nutrients tended to choose cereals with lower contents of those nutrients, while those that indicated considering positive nutrients tended to choose cereals with higher contents of those nutrients. We also analysed the relationship between the time it took to choose a cereal and the number of nutrients considered, expecting that respondents that considered more nutrients would take longer to make a decision. Indeed, validating subjects' responses, the more nutrients they reported considering during cereal choice—regardless of nutrient type—the longer it took to make a decision. This result has been found in previous research on consideration of health during food choice and the amount of time spent on decision‐making (Tuyizere and Gustafson 2023). Finally, we also found significant positive relationships between the number of positive and negative nutrients a participant considered during food choice among those participants that indicated their eating habits were ‘healthy’, but not among those that considered their eating habits were ‘unhealthy’.

One last notable limitation to this study is that nutritional information was presented in a list below the product, rather than as a formatted Nutrition Facts Panel containing full nutritional information together with an ingredient statement. The location of a nutrient on a Nutrition Facts Panel can influence its consideration during food choice. For example, information located near the centre of the nutrition panel is often viewed less than information at the top or bottom (Goldberg et al. 1999; Graham and Jeffery 2011). Additionally, the ingredient list can be perceived as a source of health and nutrition information for some consumers (Kraemer et al. 2023). Because our primary aim was to evaluate the association between consumer attention to positive or negative nutrients and the nutritional quality of food choices, we stripped out the Nutrition Facts panel formatting to focus only on the nutrients themselves. However, a future study examining the use of positive or negative nutrients in relation to their location on a formatted Nutrition Facts panel or the influence of the ingredient statement would be interesting. Notably, the nutritional information presented in our study was similar to non‐directive FOP labeling, where selected nutrient information is presented on the principal display panel in a list. Given the prevalence of FOP labels (Donini et al. 2022), our study indicates that highlighting the positive nutrients on a FOP label may help consumers make better food decisions.

In conclusion, although focusing on any nutritional information during cereal choice increased the healthfulness of cereal choices (compared with none), focusing on positive or balanced nutrients increased the healthfulness of cereal choices significantly more than focusing on negative nutrients. This study suggests that focusing nutritional recommendations on positive nutrients may have a greater influence on the nutritional quality of food choices than focusing on the negative nutrients in foods.

## Author Contributions

Conceptualisation: C.R.G., H.G. and D.J.R.; methodology: C.R.G. and H.G.; formal analysis: C.R.G. and D.J.R.; resources: C.R.G. and D.J.R.; writing – original draft preparation: D.J.R.; writing – review and editing: C.R.G., H.G. and D.J.R.; visualisation: D.J.R.; supervision: C.R.G. and D.J.R.; project administration: C.R.G.; funding acquisition: C.R.G. and D.J.R. All authors have read and agreed to the published version of the manuscript.

## Ethics Statement

This study was conducted in accordance with the Declaration of Helsinki and approved by the Institutional Review Board of the University of Nebraska‐Lincoln (IRB protocol #20201020721EX). All participants provided electronic informed consent before participating in the research.

## Conflicts of Interest

The authors declare no conflicts of interest.

## Supporting information


**Data S1:** Supporting Information.

**Table S1:** Nutrient content (per 40 g serving) and Guiding Stars rating of cereals available to participants during food choice.

## Data Availability

The data that support the findings of this study are available from the corresponding author upon reasonable request.
