# Supplementary material for: Self‐Reported Attention to Positive Versus Negative Nutrients During Breakfast Cereal Selection Is Associated With Healthier Food Choice
Source: Nutr Bull. 2025 Aug 6;50(4):666–77. doi: 10.1111/nbu.70025 (PMC12621172; doi:10.1111/nbu.70025)
Supplement: Supplementary file 1 — Data S1: Supporting Information. [file NBU-50-666-s001.docx]

Note: This is the survey instrument used in the present study: “Self-reported attention to positive versus negative nutrients during food selection is associated with healthier food choice.” It is the baseline condition for an intervention study on simple and educational point-of-decision messages on nutritional choice outcomes. The results of the intervention study are published here: <https://doi.org/10.1016/j.appet.2024.107301>.

Cereal Choice Survey (final version)

Start of Block: Informed Consent

Q1

Welcome to a study on food shopping!  

 We are interested in your experience making food choices in an online supermarket interface. You will be presented with ready-to-eat breakfast cereal products and asked to make a choice about which item you would select if faced with these cereals in real life (you can also indicate that you wouldn't select any of the items). If you shop for food for multiple people, please choose a product that you would yourself eat. You will then answer some questions about your experiences in the shopping interface, as well as a few questions about yourself. While you will not actually receive a product today or spend any money, please approach the choice as if you were going to receive the product and spend money. Think about other uses you have for your money and imagine that these choices would result in you purchasing the cereal product you select. We thank you for your participation in this research.   The study should take you around 10 minutes to complete, and you will receive $2.00 for your participation. Your participation in this research is voluntary. You have the right to withdraw at any point during the study, for any reason, and without any prejudice. This study has been approved by the University of Nebraska-Lincoln Institutional Review Board (#20201020721EX). If you would like to contact the Principal Investigator in the study to discuss this research, please message Henriette Gitungwa at hgitungwa at huskers dot unl dot edu.   By clicking the button below, you acknowledge that your participation in the study is voluntary, you are at least 19 years of age and have eaten breakfast cereal in the last 3 months and that you are aware that you may choose to terminate your participation in the study at any time and for any reason.

- I consent, begin the study (1)
- I do not consent, I do not wish to participate (2)

End of Block: Informed Consent

Start of Block: Does not consent

| 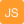 |
| --- |

Q2 As you do not wish to participate in this study, please **return** your submission on Prolific by selecting the 'Stop without completing' button.

End of Block: Does not consent

Start of Block: Record participant IDs

| 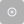 |
| --- |

Prolific ID Please enter your Prolific ID:

________________________________________________________________

End of Block: Record participant IDs

Start of Block: Demographics / screener validation

Display This Question:

If Welcome to a study on food shopping!   We are interested in your experience making food choices i... , I consent, begin the study Is Not Displayed

Instructions 1 ***NOTE TO RESEARCHER: The red text is for instructional purposes only and will not be shown to respondents.***
 
Use this block to ask the same questions as you've included in your Prolific prescreening, to validate that participants' prescreening responses are accurate. 

 If participants provide a response which is inconsistent with your prescreening criteria, you can redirect them to the 'Inconsistent screening responses' block (OPTIONAL).


For example, I want only respondents who have received a formal qualification, so have added a Branch to my Survey Flow to redirect any participants who select 'No formal qualifications' to the Inconsistent screening responses block.

Age screener What is your age (in years)?

- 18 or under (1)
- 19-24 (2)
- 25-29 (3)
- 30-34 (7)
- 35-39 (8)
- 40-44 (9)
- 45-49 (10)
- 50-54 (11)
- 55-59 (4)
- 60-64 (5)
- 65 or older (6)

End of Block: Demographics / screener validation

Start of Block: Displayed for non-eligible participant

Display This Question:

If What is your age (in years)? = 18 or under

| 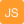 |
| --- |

Ineligible You are ineligible for this study, as you have provided information which is inconsistent with your Prolific prescreening responses. Please return your submission on Prolific by selecting the 'Stop without completing' button.

End of Block: Displayed for non-eligible participant

Start of Block: Baseline condition

Q3 Next, you will have the opportunity to make a choice of breakfast cereal. There are numerous cereals to choose from. If you do not find a cereal you would be willing to purchase, you will have the opportunity to indicate that you would not be willing to purchase any of the products. Before you select a cereal, you will make a choice about the set of cereals you want to view. You can choose to view all the cereals, or to view one of three subsets.

| Page Break |  |
| --- | --- |

Q4 Timing

First Click (1)

Last Click (2)

Page Submit (3)

Click Count (4)

| 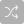 |
| --- |

Q5
What set of cereals would you like to select from?

- Cereals such as Frosted Flakes, Froot Loops, Reese's Puffs (1)
- Cereals such as Corn Flakes, Crispix, Special K (5)
- Cereals such as Cheerios, Wheat Chex, Grape Nuts (2)
- All Options (3)

| Page Break |  |
| --- | --- |

Q6 Timing

First Click (1)

Last Click (2)

Page Submit (3)

Click Count (4)

Display This Question:

If SUBSET3CEREAL = Cereals such as Frosted Flakes, Froot Loops, Reese's Puffs

| 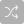 |
| --- |

Q7 Please select the box of cereal that you would most like to purchase. You may also indicate that you would not purchase any of these items if you do not find one that you are interested in purchasing.

- Image:Opt out (40)
- **Apple Jacks** Calories: 154 Fat: 1.5 g Sodium: 215 mg Fiber: 2 g Sugar: 13 gPotassium: 51mgIron: 5 mg $4.28 (1)
- **Cap'n Crunch's Crunch Berries** Calories: 162 Fat: 2 g Sodium: 292 mg Fiber: <1 g Sugar: 17 gPotassium: 65 mgIron: 8mg $4.78 (2)
- **Corn Pops** Calories: 150 Fat: 0 g Sodium: 160 mg Fiber: 0 g Sugar: 15 gPotassium: 30 mgIron: 5 mg $4.28 (34)
- **Froot Loops** Calories: 154 Fat: 1.5 g Sodium: 215 mg Fiber: 2 g Sugar: 12 gPotassium: 62 mgIron: 5 mg $4.28 (3)
- **Fruity Pebbles** Calories: 156 Fat: 2 g Sodium: 211 mg Fiber: 0 g Sugar: 13 gPotassium: 22 mgIron: 1 mg $ 4.28 (4)
- **Honey Comb** Calories: 160 Fat: 1 g Sodium: 190 mg Fiber: 1 g Sugar: 13 gPotassium: 60 mgIron: 3 mg $3.48 (8)
- **Cookie Crisp** Calories: 156 Fat: 2 g Sodium: 211 mg Fiber: 2 g Sugar: 13 gPotassium: 111 mgIron: 4 mg $4.78 (9)
- **Frosted Flakes** Calories: 141 Fat: 0 g Sodium: 205 mgFiber: 1 gSugar: 13 g Potassium: 32 mgIron: 8 mg $3.89 (15)
- **Lucky Charms** Calories: 156 Fat: 2 g Sodium: 255 mg Fiber: 2 g Sugar: 13 gPotassium: 0 mgIron: 4 mg $4.78 (27)
- **Trix** Calories: 164 Fat: 2 g Sodium: 185 mg Fiber: 1 g Sugar: 12 gPotassium: 0 mgIron:4 mg $4.78 (11)
- **Reese's Puffs** Calories: 164 Fat: 5 g Sodium: 226 mg Fiber: 2 g Sugar: 12 g Potassium: 92 mgIron: 4 mg $4.78 (14)

| Page Break |  |
| --- | --- |

Q8 Timing

First Click (1)

Last Click (2)

Page Submit (3)

Click Count (4)

Display This Question:

If SUBSET3CEREAL = Cereals such as Corn Flakes, Crispix, Special K

| 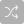 |
| --- |

Q9 Please select the box of cereal that you would most like to purchase. You may also indicate that you would not purchase any of these items if you do not find one that you are interested in purchasing.

- Image:Opt out (40)
- **Crispix** Calories: 154 Fat: 0 g Sodium: 267 mg Fiber: 0 g Sugar: 5 gPotassium: 21 mgIron: 11 mg $4.68 (38)
- **Golden Grahams** Calories:150 Fat: 1.5 g Sodium: 300 mg Fiber: 2 g Sugar: 12 gPotassium: 0 mgIron: 4 mg $4.78 (17)
- **Corn Flakes** Calories: 143 Fat: 0 g Sodium: 286 mg Fiber: 1 g Sugar: 4 gPotassium: 57 mgIron: 11 mg $4.28 (28)
- **Alpha-bits** Calories: 102 Fat: 1 g Sodium: 130 mg Fiber: 2 g Sugar: 6 gPotassium: 149 mgIron: 12 mg $3.98 (18)
- **Honey Nut Cheerios** Calories: 151 Fat: 2 g Sodium: 227 mg Fiber: 3 g Sugar: 13 g Potassium: 162 mgIron: 4 mg $4.78 (19)
- **Raisin Bran Crunch** Calories: 138 Fat: 1 g Sodium: 145 mg Fiber: 3 g Sugar: 14 gPotassium: 204 mg Iron: 1 mg $3.48 (20)
- **Rice Chex** Calories: 160 Fat: 1 g Sodium: 330 mg Fiber: 2 g Sugar: 3 gPotassium: 0 mgIron: 13 mg $4.78 (21)
- **Honey Bunches of Oats** Calories: 162 Fat: 3 g Sodium: 171 mg Fiber: 2 g Sugar: 9 gPotassium: 86 mgIron: 15 mg $4.68 (22)
- **Kellogg's Low-fat Granola w/ Raisins** Calories: 161 Fat: 2 g Sodium: 105 mg Fiber: 3 g Sugar: 12 gPotassium: 140 mgIron: 3 mg $3.68 (23)
- **Special K Red Berries** Calories: 144 Fat: 0.5 g Sodium: 256 mg Fiber: 3 g Sugar: 11 gPotassium: 82 mgIron: 11 mg $4.68 (24)
- **Oatmeal Squares** Calories: 150  Fat: 2 g Sodium: 136 mg Fiber: 3.5 g Sugar: 6 gPotassium: 143 mgIron: 12 mg $4.66 (25)

| Page Break |  |
| --- | --- |

Q10 Timing

First Click (1)

Last Click (2)

Page Submit (3)

Click Count (4)

Display This Question:

If SUBSET3CEREAL = Cereals such as Cheerios, Wheat Chex, Grape Nuts

| 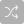 |
| --- |

Q11 Please select the box of cereal that you would most like to purchase. You may also indicate that you would not purchase any of these items if you do not find one that you are interested in purchasing.

- Image:Opt out (1)
- **Multi-Grain Cheerios** Calories: 154 Fat: 1.5 g Sodium: 154 mg Fiber: 3 g Sugar: 8 gPotassium: 185 mgIron: 18 mg $4.98 (2)
- **Great Grains Raisins Dates Pecans** Calories: 143 Fat: 3 g Sodium: 100 mg Fiber: 3 g Sugar: 9 gPotassium: 150 mgIron: 8 mg $4.48 (3)
- **Frosted Mini-Wheats Original** Calories: 140 Fat: 1 g Sodium: 6 mg Fiber: 4 g Sugar: 8 gPotassium: 107 mg Iron: 12 mg $3.48 (4)
- **Kashi Berry Fruitful** Calories: 125 Fat: 1 g Sodium: 0 mg Fiber: 4 g Sugar: 6 gPotassium: 125 mgIron: 2 mg $4.38 (5)
- **Shredded Wheat** Calories: 140 Fat: 1 g Sodium: 0 mg Fiber: 5 g Sugar: 0 gPotassium: 167 mgIron: 1 mg $3.48 (6)
- **All-Bran Buds** Calories: 98 Fat: 1 g Sodium: 267 mg Fiber: 15 g Sugar: 11 gPotassium: 382 mgIron: 4 mg $4.68 (7)
- **Cheerios** Calories: 144 Fat: 2.5 g Sodium: 195 mg Fiber: 4 g Sugar: 2 gPotassium: 256 mgIron: 13 mg $4.78 (8)
- **Wheaties** Calories: 144 Fat: 0.5 g Sodium: 267 mg Fiber: 4 g Sugar: 6 gPotassium: 156 mg Iron: 12 mg $4.98 (9)
- **Grape-Nuts** Calories: 138 Fat: 1 g Sodium: 193 mg Fiber: 5 g Sugar: 3 gPotassium: 179 mgIron: 11 mg $4.48 (10)
- **Wheat Chex** Calories: 142 Fat: 1 g Sodium: 231 mg Fiber: 5 g Sugar: 4 gPotassium: 129 mgIron: 12 mg $4.78 (11)
- **Fiber One Original** Calories: 90 Fat: 1 g Sodium: 140 mg Fiber: 18 g Sugar: 0 gPotassium: 0 mgIron: 4 mg $4.98 (12)

| Page Break |  |
| --- | --- |

Q12 Timing

First Click (1)

Last Click (2)

Page Submit (3)

Click Count (4)

Display This Question:

If SUBSET3CEREAL = All Options

| 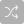 |
| --- |

Q13 Please select the box of cereal that you would most like to purchase. You may also indicate that you would not purchase any of these items if you do not find one that you are interested in purchasing.

- Image:Opt out (40)
- **Apple Jacks** Calories: 154 Fat: 1.5 g Sodium: 215 mg Fiber: 2 g Sugar: 13 gPotassium: 51 mgIron: 5 mg $4.28 (1)
- **Cap'n Crunch's Crunch Berries** Calories: 162 Fat: 2 g Sodium: 292 mg Fiber: <1 g Sugar: 17 gPotassium: 65 mgIron: 8 mg $ 4.78 (2)
- **Corn Pops** Calories: 150 Fat: 0 g Sodium: 160 mg Fiber: 0 g Sugar: 15 gPotassium: 30 mgIron: 5 mg $4.28 (34)
- **Froot Loops** Calories: 154 Fat: 1.5 g Sodium: 215 mg Fiber: 2 g Sugar: 12 gPotassium: 62 mgIron: 5 mg $4.28 (3)
- **Fruity Pebbles** Calories: 156 Fat: 2 g Sodium: 211 mg Fiber: 0 g Sugar: 13 gPotassium: 22 mgIron: 1 mg $4.28 (4)
- **Honey Comb** Calories: 160 Fat: 1 g Sodium: 190 mg Fiber: 1 g Sugar: 13 gPotassium: 60 mgIron: 3 mg $3.48 (8)
- **Cookie Crisp** Calories: 156 Fat: 2 g Sodium: 211 mg Fiber: 2 g Sugar: 13 gPotassium: 111 mgIron: 4 mg $4.78 (9)
- **Frosted Flakes** Calories: 141 Fat: 0 g Sodium: 205 mg Sugar: 13 g Fiber: 1 gPotassium: 32 mgIron: 8 mg $3.89 (15)
- **Lucky Charms** Calories: 156 Fat: 2 g Sodium: 255 mg Fiber: 2 g Sugar: 13 gPotassium: 0 mgIron:4 mg $4.78 (27)
- **Trix** Calories: 164 Fat: 2 g Sodium: 185 mg Fiber: 1 g Sugar: 12 gPotassium: 0 mgIron: 4 mg $4.78 (11)
- **Reese's Puffs** Calories: 164 Fat: 5 g Sodium: 226 mg Fiber: 2 g Sugar: 12 gPotassium: 92 mgIron: 4 mg $4.78 (14)
- **Crispix**Calories: 154Fat: 0 gSodium: 267 mg Fiber: 0 g Sugar: 5 g Potassium: 21 mgIron: 11 mg$4.68 (38) __________________________________________________
- **Golden Grahams** Calories: 150 Fat: 1.5 g Sodium: 300 mg Fiber: 2 g Sugar: 12 gPotassium: 0 mg Iron: 4 mg $4.78 (17)
- **Corn Flakes** Calories: 143 Fat: 0 g Sodium: 286 mg Fiber: 1 g Sugar: 4 gPotassium: 57 mgIron: 11 mg $4.28 (28)
- **Post Alpha-bits** Calories: 102 Fat: 1 g Sodium: 130 mg Fiber: 2 g Sugar: 6 gPotassium: 149 mgIron: 12 mg $3.98 (18)
- **Honey Bunches of Oats** Calories: 162 Fat: 3 g Sodium: 171 mg Fiber: 2 g Sugar: 9 gPotassium: 86 mgIron: 15 mg $4.68 (19)
- **Honey Nut Cheerios** Calories: 151 Fat: 2 g Sodium: 227 mg Fiber: 3 g Sugar: 13 gPotassium: 162 mgIron: 4 mg $4.78 (20)
- **Kellogg's Low-fat Granola w/ Raisins** Calories: 161 Fat: 2 g Sodium: 105 mg Fiber: 3 g Sugar: 12 gPotassium: 140 mgIron: 3 mg $3.68 (21)
- **Raisin Bran Crunch** Calories: 138 Fat: 1 g Sodium: 145 mg Fiber: 3 g Sugar: 14 gPotassium: 204 mgIron: 1 mg $3.48 (22)
- **Rice Chex** Calories: 160 Fat: 1 g Sodium: 330 mg Fiber: 2 g Sugar: 3 gPotassium: 0 mgIron: 13 mg $4.78 (23)
- **Special K Red Berries** Calories: 142 Fat: 0.5 g Sodium: 256 mg Fiber: 3 g Sugar: 11 gPotassium: 82 mgIron: 11 mg $4.68 (24)
- **Oatmeal Squares** Calories: 150  Fat: 2 g Sodium: 136 mg Fiber: 3.5 g Sugar: 6 gPotassium: 143 mgIron: 12 mg $4.66 (25)
- **Multi-Grain Cheerios** Calories: 154 Fat: 1.5 g Sodium: 154 mg Fiber: 3 g Sugar: 8 gPotassium: 185 mgIron: 18 mg $4.98 (5)
- **Great Grains Raisins Dates Pecans** Calories: 143 Fat: 3 g Sodium: 100 mg Fiber: 3 g Sugar: 9 gPotassium:150 mgIron: 8 mg $4.48 (31)
- **Frosted Mini-Wheats Original** Calories: 140 Fat: 1 g Sodium: 6 mg Fiber: 4 g Sugar: 8 g Potassium: 107 mgIron: 12 mg $3.48 (36)
- **Kashi Berry Fruitful** Calories: 125 Fat: 1 g Sodium: 0 mg Fiber: 4 g Sugar: 6 gPotassium: 125 mgIron: 2 mg $4.38 (37)
- **Shredded Wheat** Calories: 140 Fat: 1 g Sodium: 0 mg Fiber: 5 g Sugar: 0 gPotassium: 167 mgIron: 1 mg $3.48 (6)
- **All-Bran Buds** Calories: 98 Fat: 1 g Sodium: 267 mg Fiber: 15 g Sugar: 11 gPotassium: 382 mgIron: 4 mg $4.68 (39)
- **Cheerios** Calories: 144 Fat: 2.5 g Sodium: 195 mg Fiber: 4 g Sugar: 2 gPotassium: 256 mgIron: 13 mg $4.78 (13)
- **Wheaties** Calories: 144 Fat: 0.5 g Sodium: 267 mg Fiber: 4 g Sugar: 6 gPotassium: 156 mgIron: 12 mg $4.98 (12)
- **Grape-Nuts** Calories: 138 Fat: 1 g Sodium: 193 mg Fiber: 5 g Sugar: 3 gPotassium: 179 mgIron: 11 mg $4.48 (30)
- **Wheat Chex** Calories: 142 Fat: 1 g Sodium: 231 mg Fiber: 5 g Sugar: 4 gPotassium: 129 mgIron: 12 mg $4.78 (10)
- **Fiber One Original** Calories: 90 Fat: 1 g Sodium: 140 mg Fiber: 18 g Sugar: 0 gPotassium: 0 mgIron: 4 mg $4.98 (29)

| Page Break |  |
| --- | --- |

Q14 When selecting your box of cereal, which attribute(s) did you *consider*? Select all that apply.

- Brand (1)
- Calories (12)
- Fat (2)
- Sodium (10)
- Fiber (7)
- Sugar (8)
- Potassium (11)
- Iron (9)
- Price (13)
- ⊗I did not look at this information for the cereal options (14)

| Page Break |  |
| --- | --- |

Q15A What do you believe is the ***average price*** of ***Cereals such as Frosted Flakes, Froot Loops, Reese's Puffs***?

|  | 0 | 1 | 2 | 3 | 4 | 5 | 6 |
| --- | --- | --- | --- | --- | --- | --- | --- |

| Average USD () | 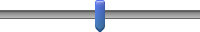 |
| --- | --- |

| Page Break |  |
| --- | --- |

Q15B How ***healthy*** do you believe ***Cereals such as Frosted Flakes, Froot Loops, Reese's Puffs*** are on average?

|  | 0 | 1 | 2 | 3 | 4 | 5 | 6 | 7 | 8 | 9 | 10 |
| --- | --- | --- | --- | --- | --- | --- | --- | --- | --- | --- | --- |

| Rate them on the following scale: “Not at all healthy”(0) to “Very healthy” (10) () | 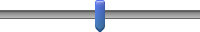 |
| --- | --- |

| Page Break |  |
| --- | --- |

Q15C How ***tasty*** do you believe ***Cereals such as Frosted Flakes, Froot Loops, Reese's Puff***” are on average?

|  | 0 | 1 | 2 | 3 | 4 | 5 | 6 | 7 | 8 | 9 | 10 |
| --- | --- | --- | --- | --- | --- | --- | --- | --- | --- | --- | --- |

| Rate them on the following scale: “Not at all tasty” (0) to “Very tasty” (10) () | 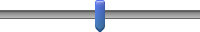 |
| --- | --- |

| Page Break |  |
| --- | --- |

Q16A What do you believe is the ***average price*** of ***Cereals such as Corn Flakes, Crispix, Special K***?

|  | 0 | 1 | 2 | 3 | 4 | 5 | 6 |
| --- | --- | --- | --- | --- | --- | --- | --- |

| Average in USD () | 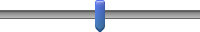 |
| --- | --- |

| Page Break |  |
| --- | --- |

Q16B How ***healthy*** do you believe ***Cereals such as Corn Flakes, Crispix, Special K*** are on average?

|  | 0 | 1 | 2 | 3 | 4 | 5 | 6 | 7 | 8 | 9 | 10 |
| --- | --- | --- | --- | --- | --- | --- | --- | --- | --- | --- | --- |

| Rate them on the following scale: “Not at all healthy”(0) to “Very healthy” (10) () | 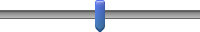 |
| --- | --- |

| Page Break |  |
| --- | --- |

Q16C How ***tasty*** do you believe ***Ce****reals such as Corn Flakes, Crispix, Special K* are on average?

|  | 0 | 1 | 2 | 3 | 4 | 5 | 6 | 7 | 8 | 9 | 10 |
| --- | --- | --- | --- | --- | --- | --- | --- | --- | --- | --- | --- |

| Rate them on the following scale: “Not at all tasty” (0) to “Very tasty” (10) () | 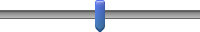 |
| --- | --- |

| Page Break |  |
| --- | --- |

Q17A What do you believe is the ***average price*** of ***Cereals such as Cheerios, Wheat Chex, Grape Nuts***?

|  | 0 | 1 | 2 | 3 | 4 | 5 | 6 |
| --- | --- | --- | --- | --- | --- | --- | --- |

| Average USD () | 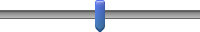 |
| --- | --- |

| Page Break |  |
| --- | --- |

Q17B How ***healthy*** do you believe ***Cereals such as Cheerios, Wheat Chex, Grape Nuts*** are on average?

|  | 0 | 1 | 2 | 3 | 4 | 5 | 6 | 7 | 8 | 9 | 10 |
| --- | --- | --- | --- | --- | --- | --- | --- | --- | --- | --- | --- |

| Rate them on the following scale: “Not at all healthy”(0) to “Very healthy” (10) () | 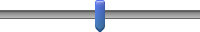 |
| --- | --- |

| Page Break |  |
| --- | --- |

Q17C How ***tasty*** do you believe ***Cereals such as Cheerios, Wheat Chex, Grape Nuts*** are on average?

|  | 0 | 1 | 2 | 3 | 4 | 5 | 6 | 7 | 8 | 9 | 10 |
| --- | --- | --- | --- | --- | --- | --- | --- | --- | --- | --- | --- |

| Rate them on the following scale: “Not at all tasty” (0) to “Very tasty” (10) () | 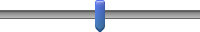 |
| --- | --- |

End of Block: Baseline condition

Start of Block: Survey

Q18 When selecting your box of cereal, which attribute(s) did you consider? Select all that apply.

- Brand (1)
- Calories (2)
- Fat (3)
- Sodium (4)
- Fiber (5)
- Sugar (6)
- Potassium (7)
- Iron (8)
- Price (9)
- ⊗I did not look at this information for the cereal options (10)

| Page Break |  |
| --- | --- |

Q19A What do you believe is the ***average price*** of ***Cereals such as Frosted Flakes, Froot Loops, Reese's Puffs?***

|  | 0 | 1 | 2 | 3 | 4 | 5 | 6 |
| --- | --- | --- | --- | --- | --- | --- | --- |

| Average in USD () | 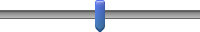 |
| --- | --- |

| Page Break |  |
| --- | --- |

Q19B How ***healthy*** do you believe ***Cereals such as Frosted Flakes, Froot Loops, Reese's Puffs*** are on average?

|  | 0 | 1 | 2 | 3 | 4 | 5 | 6 | 7 | 8 | 9 | 10 |
| --- | --- | --- | --- | --- | --- | --- | --- | --- | --- | --- | --- |

| Rate them on the following scale: “Not at all healthy”(0) to “Very healthy” (10) () | 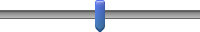 |
| --- | --- |

| Page Break |  |
| --- | --- |

Q19C How ***tasty*** do you believe ***Cereals such as Frosted Flakes, Froot Loops, Reese's Puff”*** are on average?

|  | 0 | 1 | 2 | 3 | 4 | 5 | 6 | 7 | 8 | 9 | 10 |
| --- | --- | --- | --- | --- | --- | --- | --- | --- | --- | --- | --- |

| Rate them on the following scale: “Not at all tasty” (0) to “Very tasty” (10) () | 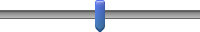 |
| --- | --- |

| Page Break |  |
| --- | --- |

Q20A What do you believe is the ***average price*** of ***Cereals such as Corn Flakes, Crispix, Special K***?

|  | 0 | 1 | 2 | 3 | 4 | 5 | 6 |
| --- | --- | --- | --- | --- | --- | --- | --- |

| Average in USD () | 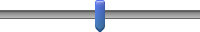 |
| --- | --- |

| Page Break |  |
| --- | --- |

Q20B How ***healthy*** do you believe ***Cereals such as Corn Flakes, Crispix, Special K*** are on average?

|  | 0 | 1 | 2 | 3 | 4 | 5 | 6 | 7 | 8 | 9 | 10 |
| --- | --- | --- | --- | --- | --- | --- | --- | --- | --- | --- | --- |

| Rate them on the following scale: “Not at all healthy”(0) to “Very healthy” (10) () | 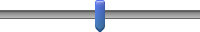 |
| --- | --- |

| Page Break |  |
| --- | --- |

Q20C How ***tasty*** do you believe ***Cereals such as Corn Flakes, Crispix, Special K*** are on average?

|  | 0 | 1 | 2 | 3 | 4 | 5 | 6 | 7 | 8 | 9 | 10 |
| --- | --- | --- | --- | --- | --- | --- | --- | --- | --- | --- | --- |

| Rate them on the following scale: “Not at all tasty” (0) to “Very tasty” (10) () | 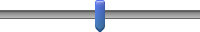 |
| --- | --- |

| Page Break |  |
| --- | --- |

Q21A What do you believe is the ***average price*** of ***Cereals such as Cheerios, Wheat Chex, Grape Nuts***?

|  | 0 | 1 | 2 | 3 | 4 | 5 | 6 |
| --- | --- | --- | --- | --- | --- | --- | --- |

| Average in USD () | 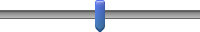 |
| --- | --- |

| Page Break |  |
| --- | --- |

Q21B How ***healthy*** do you believe ***Cereals such as Cheerios, Wheat Chex, Grape Nuts*** are on average?

|  | 0 | 1 | 2 | 3 | 4 | 5 | 6 | 7 | 8 | 9 | 10 |
| --- | --- | --- | --- | --- | --- | --- | --- | --- | --- | --- | --- |

| Rate them on the following scale: “Not at all healthy”(0) to “Very healthy” (10) () | 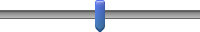 |
| --- | --- |

| Page Break |  |
| --- | --- |

Q21C How ***tasty*** do you believe ***Cereals such as Cheerios, Wheat Chex, Grape Nuts*** are on average?

|  | 0 | 1 | 2 | 3 | 4 | 5 | 6 | 7 | 8 | 9 | 10 |
| --- | --- | --- | --- | --- | --- | --- | --- | --- | --- | --- | --- |

| Rate them on the following scale: “Not at all tasty” (0) to “Very tasty” (10) () | 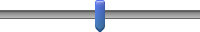 |
| --- | --- |

| Page Break |  |
| --- | --- |

| 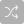 |
| --- |

Q22 Which of the following did you consider when making food choices today? *Select all that apply.*

- How much you like the taste of the foods (1)
- The healthy nutrients you can get from the foods (2)
- Avoiding unhealthy nutrients present in some foods (3)
- The impact of the foods on your current or future health (4)
- The cost of the foods in the context of your budget (5)
- Whether a food was from a brand you know/trust (8)
- Other (9) __________________________________________________

| Page Break |  |
| --- | --- |

Q23 Did you look at/consider all cereal products from the cereal category that you selected your cereal box from?

- I looked at all items and chose a cereal that I have never purchased in the past. (1)
- I looked at all of the items but ultimately chose the cereal I liked the most based on past experience (2)
- As soon as I saw the cereal that I already knew that I wanted I stopped looking at other items in the list. (3)
- I found a cereal that is satisfactory to me, and I stopped looking further (although it may not be the optimal cereal available). (4)
- I randomly chose the cereal product. (5)
- Other (please explain) (6) __________________________________________________

| Page Break |  |
| --- | --- |

| 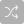 |
| --- |

Q24 Please mark how much you agree with the following statements.

|  | Disagree (1) | Somewhat disagree (2) | Neither agree nor disagree (3) | Somewhat agree (4) | Agree (5) |
| --- | --- | --- | --- | --- | --- |
| "I have a lot of knowledge about nutrition." (1) |  |  |  |  |  |
| "I understand the benefits of fiber." (2) |  |  |  |  |  |
| "I am able to distinguish healthy foods from unhealthy foods." (3) |  |  |  |  |  |

| Page Break |  |
| --- | --- |

| 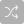 |
| --- |

Q25 Which of the following, if any, do you believe are health benefits that result from consuming dietary fiber? *Select all that apply*.

- Lowering blood glucose (1)
- Lowering cholesterol levels (2)
- Lowering blood pressure (3)
- Increasing frequency of bowel movements (4)
- Increasing mineral absorption in the intestinal tract (5)
- Reducing calorie intake (6)
- Maintaining healthy gut bacteria (7)
- Improving skin complexion (10)
- Other(please explain) (11) __________________________________________________
- ⊗None (13)

| Page Break |  |
| --- | --- |

Q26 This is a question to gauge attention. Please select "Added Sugar" from the list below.

- Calories (1)
- Fat (2)
- Sodium (3)
- Added Sugar (4)
- Protein (5)
- Fiber (6)

| Page Break |  |
| --- | --- |

Q27 Have you eaten breakfast cereal in the last 3 months?

- Yes (1)
- No (2)

| Page Break |  |
| --- | --- |

Q28 How often do you eat ready-to-eat breakfast cereal?

- Daily (1)
- Less than daily, but at least once a week (2)
- Less than weekly, but at least once a month (3)
- Less than monthly, but at least once a year (4)
- Less than yearly (5)

| Page Break |  |
| --- | --- |

Q29 I think my eating habits are:

- Healthy (1)
- Moderately healthy (2)
- Neither healthy nor unhealthy (3)
- Moderately Unhealthy (4)
- Unhealthy (5)
- I do not know (6)

| Page Break |  |
| --- | --- |

Q30 How satisfied are you with your current health status?

- Satisfied (1)
- Somewhat satisfied (2)
- Neither satisfied nor dissatisfied (3)
- Somewhat dissatisfied (4)
- Dissatisfied (5)
- Prefer not to answer (6)

| Page Break |  |
| --- | --- |

Q31  What is your age?

- 19-24 (1)
- 25-34 (2)
- 35-44 (3)
- 45-54 (4)
- 55-64 (5)
- 65 or older (6)
- Prefer not to answer (7)

| Page Break |  |
| --- | --- |

Q32 What is your gender?

- Male (1)
- Female (2)
- Other (please enter a response below) (3) __________________________________________________

| Page Break |  |
| --- | --- |

Q33

What is your current height?


If you prefer not to answer, please leave this question blank and proceed to the next page.

_______ Feet (ft) (1)

_______ Inches (in) (2)

| Page Break |  |
| --- | --- |

Q34
What is your current weight in pounds (lb)?
 

If you prefer not to answer, please leave this question blank and proceed to the next page.

_______ Pounds (lb) (1)

| Page Break |  |
| --- | --- |

Q35 What is your ethnicity? *Please select all that apply.*

- White (1)
- Hispanic or Latino (2)
- Black or African American (3)
- Native American or Alaska Native (4)
- Asian (5)
- Native Hawaiian or other Pacific Islander (7)
- Other (8)
- Prefer not to answer (9)

| Page Break |  |
| --- | --- |

Q36 What is your household income?

- Less than $20,000 (1)
- $20,000 - $39,999 (2)
- $40,000 – $59,999 (3)
- $60,000 – $79,999 (4)
- $80,000 – $99,999 (5)
- $100,000 – $119,999 (6)
- $120,000 – $139,999 (7)
- $140,000 – $159,999 (8)
- $160,000 – $179,999 (9)
- $180,000 – $199,999 (10)
- $200,000 or more (11)
- Prefer not to answer (13)

| Page Break |  |
| --- | --- |

Q37 What is the highest level of education you have completed?

- Less than high school (1)
- High school/G.E.D. (2)
- Associate's degree or some college (3)
- Bachelor's degree (4)
- Advanced degree (Master's level or higher) (5)
- Prefer not to answer (6)

End of Block: Survey
